# Utilization of and Direct Expenditure for Emergency Medical  Care in Taiwan: A Population-based Descriptive Study

**DOI:** 10.2188/jea.JE20080042

**Published:** 2009-01-30

**Authors:** Nan-Ping Yang, Yi-Hui Lee, Ching-Heng Lin, Yuan-Chang Chung, Wen-Jone Chen, Pesus Chou

**Affiliations:** 1 Community Medicine Research Center & Department and Institute of Public Health, National Yang-Ming University, Taipei, Taiwan; 2 Department of Geriatrics & Medical Research, Tao-Yuan General Hospital, Department of Health, Executive Yuan, Tao-Yuan, Taiwan; 3 The School of Nursing, Chang-Gung University, Tao-Yuan, Taiwan; 4 Department of Surgery, Medical College, National Taiwan University, Taipei, Taiwan; 5 Department of Emergency Medicine, Medical College, National Taiwan University, Taipei, Taiwan

**Keywords:** emergency, utilization, expenditure, Taiwan

## Abstract

**Background:**

We surveyed the emergency medical system (EMS) in Taiwan to provide information to policymakers responsible for decisions regarding the redistribution of national medical resources.

**Methods:**

A systematic sampling method was used to randomly sample a representative database from the National Health Insurance (NHI) database in Taiwan, during the period from 2000 to 2004.

**Results:**

We identified 10,124, 10,408, 11,209, 10,686, and 11,914 emergency room visits in 2000, 2001, 2002, 2003, and 2004, respectively. There were more males than females, and the majority of adults were younger than 50 years. Diagnose of injury/poisoning was the most frequently noted diagnostic category in emergency departments (EDs) in Taiwan. There were 13,196 (24.3%) and 2,952 (5.4%) patients with 2 and 3 concomitant diagnoses, respectively. There was a significant association between advanced age and the existence of multiple diagnoses (*P* < 0.001). With the exception of the ill-defined symptoms/signs/conditions, the two most frequent diagnoses were diseases of the circulatory system and diseases of the respiratory system in patients aged 65 years or older. On average, treatment-associated expenditure and drug-associated expenditure in Taiwan EDs averaged NT$1,155 ($35.0) and NT$190 ($5.8), respectively, which was equal to 64.5% and 10.6% of the total ED-associated cost. General ED medical expenditure increased with patient age; the increased cost ratio due to age was estimated at 8% per year (*P* < 0.001).

**Conclusions:**

The frequency of major health problems diagnosed at ED visits varied by age: more complicated complaints and multiple diagnoses were more frequent in older patients. In Taiwan, the ED system remains overloaded, possibly because of the low cost of an ED visit.

## INTRODUCTION

The emergency department (ED) is a vital component of the health care “safety net”.^1^ It is important for a developing country to establish a comprehensive emergency medical system; however, ED overcrowding and ambulance diversion have become increasingly significant national problems over the last decade.^2^ A recent survey revealed that 62% to 91% of hospital ED directors report that overcrowding is a problem;^3^^,^^4^ it is a problem not limited to large urban centers, or academic and teaching hospitals.^4^ Overcrowding in ED treatment areas threatens public health by compromising patient safety and jeopardizing the reliability of the entire emergency care system.^1^ One report noted that the problem of overcrowding was due to inappropriate use of emergency services by those without urgent conditions, that it is probably cyclical, and that it requires no specific policy response.^2^ In addition, the aging of the population has contributed to the increased number of visits, because older patients have higher visit rates.^5^ As compared with younger persons, older adults use emergency services at a higher rate, their visits are more urgent, and their stays in the emergency department are longer.^6^ As the elderly population grows, the emergency medical system (EMS) must prepare for an increase in the number of older adults seeking treatment. In the United States, the average rate of EMS utilization by the elderly (persons aged ≥65 years) was more than 4 times that of younger patients.^7^ Despite these problems in the EMS, the effort to improve the effectiveness of the EMS is justified because emergency medicine offers many tools for improving public health.^8^ In 1994, the average number of daily ED visits nationwide in Taiwan was 11,888. This increased to 13,634 in 1996, 14,911 in 1998, 16,943 in 2000, 18,085 in 2002, and 18,823 in 2004. However, the numbers of accredited hospitals in Taiwan decreased from 828, to 773, 719, 669, 610, and 590, respectively, in the same years.^9^ ED overcrowding has been a continuing problem in Taiwan, as has the uneven distribution of ED visits. In 2003, for example, there were 24 tertiary medical centers in Taiwan, and the average number of ED visits at these centers was 187.5 cases per day—6.7 times higher than the national average.^10^ It is clear, therefore, that a nationwide descriptive study of EMS utilization is needed in Taiwan. We conducted an epidemiologic survey of EMS in Taiwan in order to provide sufficient information for policymakers to make decisions regarding the redistribution of national medical resources.

## METHODS

### Data gathering

Taiwan launched a single-payer National Health Insurance (NHI) Program on March 1, 1995. As of 2007, 22.60 million of Taiwan’s 22.96 million-person population were enrolled in this program. Foreigners in Taiwan are also eligible to enroll this program. Therefore, Taiwan’s NHI Plan has accumulated data on 23.75 million people and their claims, the largest set of such data in the world. In order to respond to current and emerging health issues rapidly and effectively, the National Health Research Institute (NHRI), in cooperation with the National Health Insurance Bureau (NHIB), has established an NHI research database. The NHRI safeguards the privacy and confidentiality of subjects and routinely transfers health insurance data from the NHIB to health researchers for analysis, with the aim of improving the health of Taiwan’s citizens.

We used a systematic sampling method to randomly sample a representative database from the entire NHI research database. The size of the subset for each month was determined by the ratio of the amount of the data for that month to that for the entire year. Then, systematic sampling was performed for each month, and a representative subset was randomly chosen. This sampling database was constructed by combining the subsets of the 12 months. The sampling database for “ambulatory care expenditures by visits” was constructed first, and then analysis of the related observations for “details of ambulatory orders” was performed. The sampling database for “ambulatory care expenditures by visits” was 0.2% of the size of the entire database.

### Quality control of medical services and coding in Taiwan’s NHI system

The NHIB has established a uniform system to control the quality of medical services and codes. Under the rules of this system, if medical services provided to beneficiaries by the contracted medical care institutions are judged by the Professional Peer Review Committee to be incompatible with the provisions of the NHI Act, such expenses must be borne by the contracted medical care institutions. In cases where drugs, laboratory tests, or diagnostic examinations are provided by third-party medical care institutions in accordance with the physician's instructions, and the insurer—after conducting an examination according to established rules—decides not to pay the benefits because the physician's instructions were improper, the expenses incurred must be borne by the medical institution where the physician practices. When disputes arise, there is a Disputes Settlement Board, established under the National Health Insurance Act, which settles disputes arising from cases approved by the insurer and raised by the insured, group insurance applicants, or the contracted medical care institutions.

### Inclusion Criteria

Emergency cases served by the NHI systems were recorded within the database of “ambulatory care expenditures by visits,” and were classified into 38 case types. One case type concerned with emergency medical services and another concerned with “emergency dentist visits” do not concern the present research. Cases during the period of 2000 to 2004 that were classified as a “medical emergency” were included in the analysis.

### Statistical analyses

Descriptive statistics are presented as numbers of cases, percentages, and means with standard deviation (SD). Pearson’s chi-square (*χ*^2^) test, analysis of variances (ANOVA), and post-hoc ANOVA (Scheffé test) were used to evaluate the significance of differences; the Mantel-Haenszel (M-H) *χ*^2^ test was used to examine trends. All statistical calculations were performed using the Statistical Package for Social Sciences for Windows (SPSS for Windows 13.0).

## RESULTS

### Distribution and characteristics of emergency visits

Based on the above inclusion criteria, there were 10,124, 10,408, 11,209, 10,686, and 11,914 cases identified in 2000, 2001, 2002, 2003, and 2004, respectively. As calculated from the annual ED visits, the sampling rates were 2.03%, 2.00%, 2.01%, 2.05%, and 2.04% respectively.^11^ Table 1 shows the detailed sex and age distributions of the 54,341 randomly sampled subjects who visited the ED during 2000–2004 in Taiwan. There were more males than females, and the majority of adults were younger than 50 years. The distribution of final principal diagnoses at all visits was determined by using the International Classification of Diseases, Ninth Revision, Clinical Modification (ICD-9-CM), as shown in Table 2. Diagnoses of injury or poisoning; ill-defined symptoms, signs, or conditions; and diseases of the respiratory system were the 3 most common diagnostic categories in emergency units in Taiwan. 

**Table 1.** Characteristics of patients from sampled emergency departments in Taiwan, 2000–2004

	2000	2001	2002	2003	2004	2000–2004
						
	Cases (%)	Cases (%)	Cases (%)	Cases (%)	Cases (%)	Cases (%)
Sex^*^						
Male	5285 (52.2)	5580 (53.6)	6000 (53.5)	5737 (53.7)	6282 (52.7)	28884 (53.2)
Female	4734 (46.8)	4699 (45.1)	5095 (45.5)	4829 (45.2)	5555 (46.6)	24912 (45.8)
Age (yrs)						
0–9	2326 (23.0)	2186 (21.0)	2406 (21.5)	1998 (18.7)	2283 (19.2)	11202 (20.6)
10–19	1045 (10.3)	1122 (10.8)	1198 (10.7)	1086 (10.2)	1156 (9.7)	5607 (10.3)
20–29	1639 (16.2)	1889 (18.1)	1936 (17.3)	1996 (18.7)	2147 (18.0)	9605 (17.7)
30–39	1317 (13.0)	1306 (12.5)	1395 (12.4)	1300 (12.2)	1543 (13.0)	6861 (12.6)
40–49	1112 (11.0)	1146 (11.0)	1201 (10.7)	1241 (11.6)	1418 (11.9)	6118 (11.3)
50–59	798 (7.9)	766 (7.4)	888 (7.9)	870 (8.1)	1097 (9.2)	4419 (8.1)
60–69	793 (7.8)	794 (7.6)	845 (7.5)	821 (7.7)	862 (7.2)	4115 (7.6)
70–79	792 (7.8)	795 (7.6)	909 (8.1)	931 (8.7)	914 (7.7)	4341 (8.0)
80+	302 (3.0)	401 (3.9)	431 (3.8)	443 (4.1)	494 (4.1)	2071 (3.8)

Total	10,124(18.6)	10,408(19.2)	11,209(20.6)	10,686(19.7)	11,914(21.9)	54,341(100.0)

^*^ There are missing data in 2000 (n = 105), 2001 (n = 129), 2002 (n = 114), 2003 (n = 120), and 2004 (n = 77).

The number of males was significantly higher than the number of females (*P* < 0.001, Pearson’s *χ*^2^ test)

**Table 2.** Distribution of patient disease codes in sampled emergency departments in Taiwan, 2000–2004

Diagnosis	ICD-9-CM Codes	No. of Cases^*^	Proportion
(Disease Category)	(Range)	(n = 54,341)	(%)
I. Infectious and parasitic diseases	001–139, 7713, 320–322	1827	3.4
II. Neoplasms	140–208	935	1.7
III. Other tumors	210–239	102	0.2
IV. Endocrine, nutritional, metabolic, and immune disorders	240–289	2754	5.1
V. Mental disorders	290–319	1309	2.4
VI. Diseases of the nervous system	323–359	551	1.0
VII. Diseases of the sense organs	360–389	1619	3.0
VIII. Diseases of the circulatory system	390–459	3751	6.9
IX. Diseases of the respiratory system	460–466, 470–478,	13210	24.3
	480–519		
X. Diseases of the digestive system	520–579	10741	19.8
XI. Diseases of the genitourinary system	580–629	3442	6.3
XII. Complications of pregnancy, child birth, and the puerperium	630–676	393	0.7
XIII. Diseases of the skin and subcutaneous tissue	680–709	1737	3.2
XIV. Diseases of the musculoskeletal and connective tissue	710–739	1596	2.9
XV. Congenital abnormalities	740–759	49	0.1
XVI. Certain conditions originating in the perinatal period	760–7712, 7714–779	39	0.1
XVII. Symptoms, signs, and ill-defined conditions	780–799	14283	26.3
XVIII. Injury and poisoning	800–999	14330	26.4
XIX. Supplementary classification of external causes of injury and poisoning	E800–E999	624	1.1
XX. Supplementary classification of factors influencing health status and	V00–V82	140	0.3
contact with health services			

*: There were 13,196 patients (24.3%) with 2 distinct diagnoses and 2952 patients (5.4%) with 3 distinct diagnoses.

**Figure 1.**
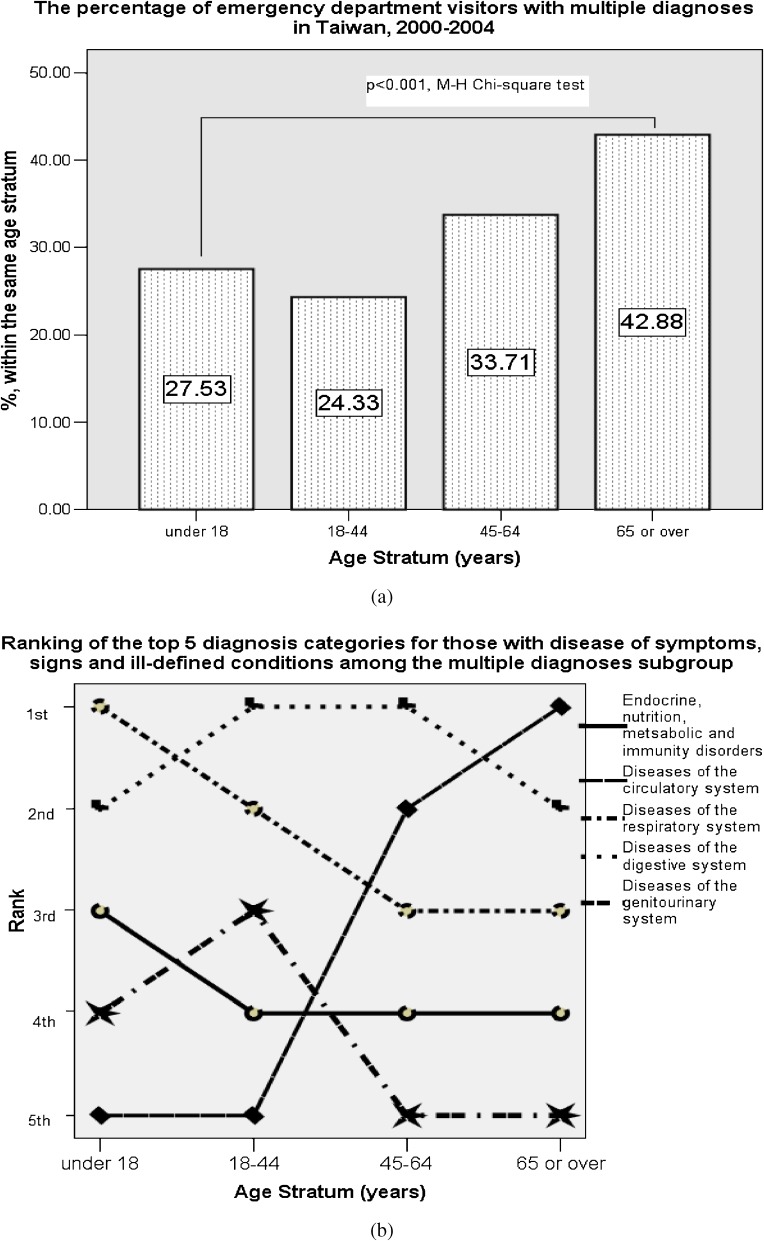
Distribution of multiple diagnoses among emergency department patients in Taiwan, 2000–2004. (a) 16,148 patients (29.7%) received multiple diagnoses from the 20 disease categories. There was a significant difference between the youngest and oldest strata in the proportion of patients with multiple diagnoses. (b) Among these patients with multiple diagnoses, the most common diagnosis was ill-defined symptoms, signs, or conditions, which was coded in 55.9% of cases. For those in this subgroup, the ranks of the other diagnosis categories are shown by patient age

There were 13,196 (24.3%) and 2952 (5.4%) cases noted with 2 and 3 concomitant categorical diagnoses, respectively. These emergency visitors with multiple diagnoses were analyzed and the results are shown in Figure 1A and B . The proportion of patients with multiple diagnoses significantly increased with age (*P* < 0.001, by the M-H *χ*^2^ test), and subjects aged 65 years or older had the highest percentage of multiple diagnoses (43%; Figure 1A). The most common diagnostic category among this subgroup was that of ill-defined symptoms, signs, or conditions, which was noted in 55.9% of cases with multiple diagnoses. Among these cases, the other diagnoses were calculated and ranked (Figure 1B). The most common second diagnosis was diseases of the digestive system (diagnosis category X), followed by diseases of the respiratory system (diagnosis category IX). The rank of diseases of the circulatory system (diagnosis category VIII) markedly increased with age, and was the most common second diagnosis of patients aged 65 years or older, along with a diagnosis of ill-defined symptoms, signs, or conditions.

### Patterns of disorder by age

In the present study, there were 14,842, 21,636, 9,365, and 8,498 cases in the age strata of under 18, 18–44, 45–64, and 65 years or older, respectively. The 10 most common disease categories among these different age groups were calculated and are shown in Table 3. In cases under 18 years of age the greatest number of visits was due to diseases of the respiratory system, followed by injury/poisoning. With the exception of the diagnostic category of ill-defined symptoms, signs, or conditions, the 2 most common disorders were injury/poisoning and diseases of the digestive system in those aged 18–44 years and 45–64 years, and diseases of the circulatory system and diseases of the respiratory system in those aged 65 or older. The distributions of the 10 most common diagnostic categories among the 4 age strata were significantly different (*P* < 0.001, Pearson’s *χ*^2^ test). In particular, we noted a significant increase with age in 4 diagnostic categories: endocrine/immune associated disorders; diseases of the circulatory system; diseases of the genitourinary system; and ill-defined symptoms, signs, or conditions (*P* < 0.01, M-H *χ*^2^ test).

**Table 3.** The 10 most common diagnoses among Taiwanese emergency patients by age stratum, 2000–2004

		Under 18		18–44		45–64		65 or over		
Age (years)		(n = 14,842)		(n = 21,636)		(n = 9,365)		(n = 8,498)		
					
		No.	%, within	No.	%, within	No.	%, within	No.	%, within	*χ*^2^ test
Diagnostic Category			the same		the same		the same		the same	
			age		age		age		age	
			stratum		stratum		stratum		stratum	
(a)	Infectious and parasitic	910	6.1	368	1.7	212	2.3	337	4.0	*P* < 0.001
	diseases									
(b)	Endocrine, nutritional,	306	2.1	641	3.0	761	8.1	1046	12.3	*P* < 0.001
	metabolic, and immune									
	disorders									
(c)	Diseases of the sense	606	4.1	470	2.2	296	3.2	247	2.9	*P* < 0.001
	organs									
(d)	Diseases of the circulatory	42	0.3	493	2.3	1174	12.5	2042	24.0	*P* < 0.001
	system									
(e)	Diseases of the	6777	45.7	3686	17.0	1245	13.3	1502	17.7	*P* < 0.001
	respiratory system									
(f)	Diseases of the digestive	3144	21.2	4316	19.9	1831	19.6	1450	17.1	*P* < 0.001
	system									
(g)	Diseases of the	272	1.8	1443	6.7	824	8.8	903	10.6	*P* < 0.001
	genitourinary system									
(h)	Diseases of the skin and	539	3.6	698	3.2	321	3.4	179	2.1	*P* < 0.001
	subcutaneous tissue									
(i)	Symptoms, signs, and	3173	21.4	5584	25.8	2786	29.7	2740	32.2	*P* < 0.001
	ill-defined conditions									
(j)	Injury and poisoning	3297	22.2	7159	33.1	2432	26.0	1442	17.0	*P* < 0.001

^*^: 24.3% and 5.4% of patients had 2 or 3 diagnoses, respectively, and these patients were distributed among the different age strata. The sum of the percentages of different diagnosis within the same age strata may exceed 100%.

### Medical expenditure in emergency departments

The direct medical expenditure (ie, cost) on emergency medicine provision in Taiwan was also investigated (Table 4). On average, a visit to a Taiwan ED cost NT$1792 (US$54.3) for insurers. The total cost increased significantly from 2000 to 2004; the annual rate of increase was estimated at 4.9% (*P* < 0.001, examined by simple linear regression). Diagnosis-associated expenditure is managed by the Taiwan government. In addition, the average treatment-associated expenditure and drug-associated expenditure in Taiwan EDs were NT$1,155 ($35.0) and NT$190 ($5.8), which represented 64.5% and 10.6% of the total ED-associated cost, respectively.

**Table 4.** Medical expenditure in emergency departments in Taiwan, 2000–2004

	Diagnosis Cost (NT$)	Treatment Cost (NT$)	Drug Cost (NT$)^*^	Total Cost (NT$)
				
	Mean (S.D.)	Mean (S.D.)	Mean (S.D.)	Mean (S.D.)
Coded Year				
2000 (group 1)	335.5 (125.5)	1060.9 (2736.4)	216.2 (1959.4)	1651.0 (3554.4)
2001 (group 2)	375.4 (81.8)	1092.1 (2467.6)	203.0 (1683.9)	1708.9 (3187.4)
2002 (group 3)	405.4 (70.6)	1084.5 (2117.9)	184.6 (1687.2)	1712.8 (2845.7)
2003 (group 4)	410.7 (70.0)	1308.5 (2510.6)	193.0 (1901.5)	1954.4 (3336.5)
2004 (group 5)	494.0 (127.3)	1219.5 (2329.9)	156.9 (710.7)	1913.9 (2612.6)

*2000–2004 overall*	407.1 (112.1)	1155.2 (2434.0)	189.6 (1633.2)	1792.1 (3109.6)†
ANOVA	*P* < 0.001	*P* < 0.001	n.s.	*P* < 0.001
*Post-hoc test*	1–2, 1–3, 1–4, 1–5,	1–4, 1–5, 2–4, 2–5,		1–4, 1–5, 2–4, 2–5,
*(difference between*	2–3, 2–4, 2–5,	3–4, 3–5		3–4, 3–5
*groups)*	3–4, 3–5, 4–5			

n.s.: not significant

^*^: in total, there were 2390 patients (4.4%) who used emergency resources without receiving pharmacologic treatment.

†: on average, the out-of-pocket cost for residents enrolled in Taiwan’s NHI programs was NT$207.8 ± 129.3 (about 11.6% of the total cost) when using emergency services.

Note: the currency conversion rate is US$1 = NT$33.

**Figure 2.**
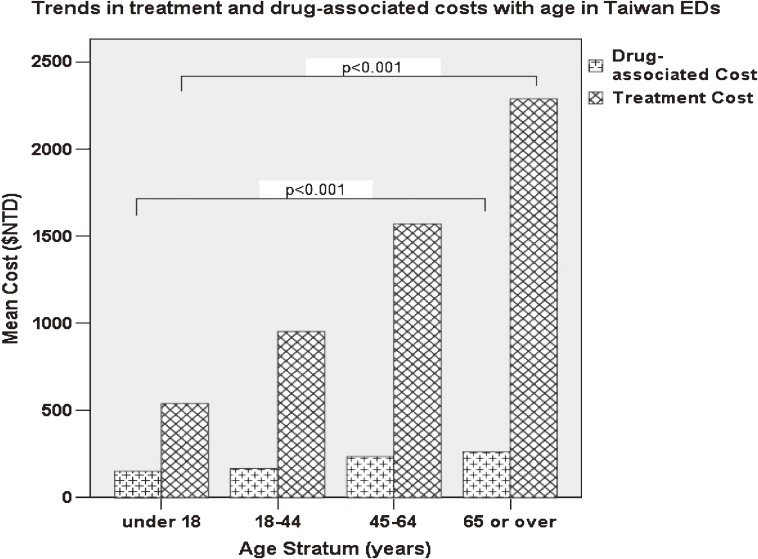
The cost of treatment was significantly positively associated with age (*P* < 0.001, simple linear regression). The cost of medication also increased with age (*P* < 0.001, simple linear regression), but the difference was significant only between patients aged 18–44 years and those aged 4564 years (examined by ANOVA post-hoc test)

To investigate the effect of age on ED medical expenditure, treatment-associated cost and drug-associated cost were calculated for the different age strata (Figure 2). Treatment-associated cost markedly increased with age, and the increase in cost due to age was estimated at 8% per year (*P* < 0.001, tested by simple linear regression). Although drug-associated cost also significantly increased with age, the difference was noted only in patients older than 44 years (measured by post-hoc ANOVA).

## DISCUSSION

The International Quality Indicator Project (IQIP) of the Center for Performance Science (CPS) compares the performance of various indicators of healthcare. Data are supplied by individual organizations and aggregated by CPS. In 2004, 676 facilities, including 239 international (non-US) facilities, were included in the aggregated, quarterly report. The hospitals located outside of the United States were in Europe (United Kingdom, Belgium, Netherlands, Germany, Austria, and Portugal) and Asia (Taiwan and Singapore). According to the acute-care indicators report for the fourth quarter of 2004, aggregated descriptive statistics showed that unscheduled returns to the ED within 24 hours and 48 hours represented 0.99% and 1.51% of ED visits in the United States, 2.71% and 4.16% of visits in Europe, and 1.33% and 2.15% of visits in Asia. Interestingly, these indicators of unscheduled revisits in ED were positively associated with the amount of ED utilization. The reported denominators (ie, ED visitors per hospital) were approximately 2,400, 5,200, and 8,900 cases per month in the United States, Asia, and Europe, respectively.^12^

According to data from the 2002 US National Hospital Ambulatory Medical Care Survey (NHAMCS), children (<19 years) and seniors (65+ years) represented 27% and 15% of all ED visits, respectively.^13^ The present study revealed a similar distribution in Taiwan (27.3% and 15.6% of all ED visitors were aged <18 and ≥65 years, respectively). A report based on data from the 2005 US NHAMCS showed that an estimated 115.3 million visits were made to hospital EDs, ie, approximately 39.6 visits per 100 persons, and that there were an estimated 41.9 million injury-related visits, or 14.4 visits per 100 persons. In addition, visit rates increased by 31 percent from 1995 to 2005, with substantial increases in patients aged 22–49 years, 50–64 years, and 65 years or older.^14^ Using data from the 2002 US National Health Care Survey, 76 million nonfatal acute injuries received initial medical attention at EDs (46.2%), physician offices (47.8%), and outpatient departments (6.0%); among ED visits for injuries, the distribution of patients in different age strata were 27%, 30%, 31%, and 12% for patients aged <18, 18–34, 35–64, and >64 years, respectively.^15^ In an analysis of the category of injury/poisoning, young and middle-aged adults were the majority of those treated in EDs for injuries (33.1% and 26.0% in the age groups of 18–44 and 45–64 years, respectively).

In Taiwanese EDs, the number of patients diagnosed with multiple co-morbidities (>2 major diagnostic categories) markedly increased with age, and treatment of the elderly required more attention and medical resources. In a study sampling 95,173 residents of Canada aged 65 or older, the presence of 2 co-morbidities (cardiovascular diseases and digestive diseases) was associated with increased ED use; the adjusted rate ratios were 1.41 (95% CI, 1.39–1.44) and 1.66 (95% CI, 1.64–1.68), respectively.^16^ In the United Kingdom, an investigation concluded that pressure on emergency care was associated with a disproportionate increase in the number of elderly patients. Between 1990 and 2004, the total number of patients had increased by 54%, with a disproportionate increase of 198% in patients aged over 70 years. They also found that the time required to manage patients increases with age, and that older patients required 9.8 times more emergency bed days.^17^ Perhaps this situation is likely to occur in Taiwan because an increase in the proportion of elderly patients treated in EDs has been noted in our survey: the percentage of elderly patients, ie, those aged 70 or older, increased from 10.8% to 12.8% from 2000 to 2004.

In an investigation by the 1998 State of California’s Office of Statewide Health Planning and Development, it was found that the overall average cost per visit was $192 for trauma EDs and $126 for non-trauma EDs.^18^ A survey performed at 6 community hospitals in the US state of Michigan revealed that for all emergency department visits, the average direct cost was $166.3; furthermore, the average direct costs were $46.0 for non-urgent visits, $127.2 for semi-urgent visits, and $280.5 for urgent visits.^19^ As compared with the results of the above studies, the average cost of ED treatment in Taiwan was found to be much lower: NT$1792 ($54.3). Taiwan’s health insurance program, which began in 1995, is a single-insurer system with a cost containment policy that mandates a fixed co-payment by the insured person for ambulatory medical services. This policy probably resulted in the increase in daily ED utilization (158% from 1994 to 2004).^9^

The lower medical cost for ED treatment in Taiwan reflects the fact that a lower percentage of total medical insurance expenditure was spent on emergency medical care. Data from the National Health Insurance of Taiwan on annual total medical insurance costs and ED-associated costs indicate that the percentage of the total NHI expenditure that was spent on NHI medical emergency care was 2.9% in each of the years from 2000 to 2003 and 3.1% in 2004.^11^ In another retrospective study using 2002 data from the US state of Oregon’s Medicaid program, monthly ED-associated expenditures averaged $12.63 (95% confidence interval, 12.50 to 12.77) per person, representing 6.8% of total medical expenditure.^20^ The average National Health Expenditure (NHE) per capita was $6120 in USA and only $939 in Taiwan. If NHE is compared to Gross Domestic Product (GDP), the NHE/GDP ratio was lower in Taiwan (6.2%) than in the United States (15.2%), Germany (10.9%), France (10.4%), Portugal (10.0%), Netherlands (9.2%), United Kingdom (7.9%), and even Japan (8.0%) in 2003.^21^ Taken together, these findings show that although the number of ED visits in Taiwan is high, the cost of ED treatment is lower than in other countries.

In Australia, a retrospective analysis of 62,495 probabilistically linked emergency hospital admissions and death records showed that hospital and ED overcrowding was associated with increased mortality.^22^ Commonly studied causes of crowding included nonurgent visits, “frequent-flyer” patients, influenza season, inadequate staffing, inpatient boarding, and hospital bed shortages. Commonly studied effects of crowding included patient mortality, transport delays, treatment delays, ambulance diversion, patient elopement, and financial hardship. Commonly studied solutions of crowding included additional personnel, observation units, hospital bed access, nonurgent referrals, ambulance diversion, destination control, crowding measures, and queuing theory.^23^ A consideration of these options from the latest systematic review would be helpful in assisting the government of Taiwan’s efforts to initiate essential studies, organize all associated facilities, and formulate a comprehensive policy for EMS.

This study did possess some limitations. First, the study used a database that was developed from the NHI database in Taiwan, within which all medical data (including final diagnoses, treatments, and medications) were entered by medical personnel and payments for all procedures were determined by the medical facilities. The accuracy of diagnosis and quality of medical services was monitored by NHIB, but there was no additional confirmation of these data. Another limitation of the study was that fewer subjects were available for sampling in 2003, because ED visits decreased by 40% to 50% during the SARS epidemic, a situation that persisted for 3 months after the end of the epidemic.^24^^,^^25^ Finally, the value of the Taiwanese currency fluctuated considerably during the years from 2000 to 2004, which makes it more difficult to interpret the currency conversions we have utilized.

## CONCLUSIONS

The principal diagnoses for patients visiting EDs varied by age. In addition, overloading of the ED system in Taiwan is still evident, possibly because of the low cost of an ED visit. Implementing the widespread primary care reforms necessary to meaningfully reduce ED utilization will be complex and expansive, although they are likely to benefit both patients and the health system.^26^ Complicated complaints and multiple diagnoses occurred more frequently in the elderly, and this should be considered when policymakers plan changes to Taiwan’s emergency system and resource allocation. Strategies to improve continuity of care may result in lower ED use and reduced health care costs. Such strategies might prove superior to current managed care policies that attempt to control costs by denying access to emergency care.^27^ Recently, a group-visit model has been studied and deemed effective in improving patient and physician satisfaction, quality of care, quality of life, and in decreasing visits to emergency departments and specialists.^28^ Therefore, we believe that additional public education regarding preventive medicine and increased activities geared toward community health promotion would prove suitable and effective in decreasing ED crowding in Taiwan.
